# An evaluation of a tuberculosis case-finding and treatment program among Syrian refugees—Jordan and Lebanon, 2013–2015

**DOI:** 10.1186/s13031-019-0213-1

**Published:** 2019-07-09

**Authors:** Andrew T. Boyd, Susan T. Cookson, Ibrahim Almashayek, Hiam Yaacoub, M. Saiful Qayyum, Aleksandar Galev

**Affiliations:** 1 Epidemic Intelligence Service, Centers for Disease Control and Prevention, Atlanta, GA USA; 2 Division of Global Health Protection, Center for Global Health, Centers for Disease Control and Prevention, Atlanta, GA USA; 3 Current affiliation: Division of Global HIV and TB, Center for Global Health, Centers for Disease Control and Prevention, 1600 Clifton Road NE, MS E-04, Atlanta, GA 30329 USA; 4 National Tuberculosis Program, Amman, Jordan; 5 National Tuberculosis Program, Beirut, Lebanon; 6 International Organization for Migration, Amman, Jordan

**Keywords:** Refugees, Tuberculosis, Syria, Jordan, Lebanon, Case-finding, Contact investigations

## Abstract

**Background:**

The displacement crisis in Syria poses challenges for tuberculosis (TB) control across the region. Since 2012 in Jordan and 2013 in Lebanon, the International Organization for Migration (IOM) has supported the National TB Program (NTP) in detecting and treating TB among Syrian refugees. In December 2016, IOM asked US Centers for Disease Control and Prevention (CDC) staff to evaluate its program of support to Jordan and Lebanon’s NTPs for TB control among Syrian refugees. This manuscript focuses on case-finding, including contact investigations, and treatment components of the IOM program during 2013–2015 in Jordan and 2015 in Lebanon.

**Methods:**

The evaluation consisted of a retrospective review of de-identified Jordan and Lebanon line lists of TB cases and of investigated contacts (Lebanon only). Syrian refugee TB cases were categorized by sex, age group (age < 5 years, 5–14 years, ≥15 years), TB type (pulmonary versus extra-pulmonary), and additionally in Jordan, by refugee camp status (residence in versus outside a refugee camp), to evaluate differences in treatment completion and contact investigation.

**Results:**

In Jordan, Syrian refugee cases represented 24.4% of TB cases in 2013, when Syrian refugees made up 6.8% of the country’s population, and 13.8% of TB cases in 2015, when Syrians made up 8.3% of the total population. In Lebanon in 2015, Syrian refugee cases represented 21.4% of TB cases, when Syrians made up 20.1% of the total population. In Jordan, the proportion of Syrian TB cases residing in refugee camps (29.3%) was higher than the proportion of Syrians refugees residing in camps (17.1%). Of Syrian TB cases in 2015, 94.8% in Jordan and 87.8% in Lebanon completed treatment. In Lebanon, among Syrian TB cases with household contacts listed, contact investigation was completed for 77.8% of cases.

**Conclusion:**

IOM’s program of NTP support provides critical TB services for Syrian refugees with high treatment completion rates. More community and health practitioner outreach for enhanced active case finding among community-based Syrian refugees in Jordan may improve TB case detection in populations outside of refugee camps. Thorough contact investigations need continued emphasis, including completely recording investigations in both countries, to find active TB cases.

## Background

The ongoing conflict in Syria has had far-reaching, devastating health consequences, with 400,000 violence-associated deaths, and 11 million people displaced since 2011 [1, 2]. Combatants have specifically targeted health infrastructure, with 382 reported attacks on facilities and 60% of infrastructure non-functional by June 2016 [3, 4]. The public health consequences are particularly problematic for those with diseases requiring treatments of long duration, like tuberculosis (TB). Previous studies of crisis-affected populations found elevated TB incidence rates and delayed TB treatment, compared with reference populations [5, 6].

The loss of health services and the displacement of Syrians pose challenges for TB control in neighboring countries, including Jordan and Lebanon. In 2013, Jordan’s TB incidence rate was 6 per 100,000 population, and in Lebanon, it was 16 per 100,000 population [7], but progress in further decreasing incidence then stalled [8, 9]. Displaced Syrians living with TB may not complete treatment, and this disruption may contribute to clinical demise, in addition to ongoing TB transmission and to drug resistance [10]. Syrian refugees living inside camps or informal settlements, which can have high population densities and poor ventilation, may be at an elevated risk of TB exposure. Finally, National TB Programs (NTPs) in Jordan and Lebanon face challenges in funding, human resources, and access in providing TB case-finding and treatment services to arriving Syrian refugees.

Recognizing the additional TB burden and the potential elevated risk of TB transmission in these host countries as the influx of Syrians grew, the International Organization for Migration (IOM) began a program of support to each country’s NTP in detecting and treating TB among Syrian refugees in 2012 in Jordan and in 2013 in Lebanon. IOM offered enhanced support in Lebanon through a Global Fund Emergency Grant in 2015. In Jordan, this support was realized through a collaboration with United Nations High Commissioner for Refugees (UNHCR), the World Health Organization (WHO), and United States Centers for Disease Control and Prevention (CDC) [11]. In Lebanon, IOM and the NTP followed a similar strategy. The program goal was to reduce TB transmission, morbidity, and mortality among Syrian refugees in these countries. Among other objectives, the program emphasized raising awareness and knowledge of TB treatment services among both Syrian refugees and health care workers, increasing active TB screening, and maximizing treatment success among Syrian refugees, largely through community health workers and other community outreach (Table 1).

**Table 1:** Description of International Organization for Migration (IOM) support program to the Jordan and Lebanon National Tuberculosis Programs (NTPs) for TB control among Syrian refugees—Jordan and Lebanon, 2013–2015

Program goal: Reduce susceptible and resistant tuberculosis transmission, morbidity, and mortality among Syrian refugees	
Objectives:	
1) Increase TB awareness and knowledge of TB treatment services among Syrian refugees and health care workers	
2) Increase TB screening among Syrian refugees	
3) Increase TB diagnosis among Syrian refugees	
4) Maximize treatment success among Syrian refugees	
5) Support the development and implementation of national guidelines for effective management of latent TB infection (LTBI)	
Resources/Activities in Jordan, 2013–2015:	
• Engage community health workers to provide awareness sessions about TB disease and TB treatment services in refugee camps and to community organizations where Syrian refugees live	
• Provide symptom screening at international border entry point (until 2014) for arriving Syrian refugees	
• Employ physicians to provide assessment of presumed Syrian refugee TB cases where Syrian refuges live using mobile medical unit	
• Provide funds for culture and drug sensitivity testing, if indicated, for Syrian refugees	
• Provide funds for TB medications for Syrian refugees	
• Engage community health workers to provide directly observed therapy (DOT) to Syrian refugee TB cases in camps and contract community-based organizations (CBOs) to provide it where Syrian refugees live in the community	
• Provide funds for transportation for contacts to come to NTP clinic for contact investigations among Syrian refugees	
Resources/Activities in Lebanon, 2015	
• Engage community health workers to provide mass TB symptom screenings in informal settlements and collective shelters where Syrian refugees live	
• Provide funds for transportation to refer presumed Syrian refugee TB cases to NTP clinics for physician assessment	
• Provide funds for culture and drug sensitivity testing, if indicated, for Syrian refugees	
• Provide funds for TB medications for Syrian refugees	
• Support salary for DOT coordinators (nurses) to provide DOT to Syrian refugee TB cases	
• Provide funds for transportation for contacts to come to NTP clinic for contact investigation among Syrian refugees	
• Provide funds for diagnostic workup for contacts among Syrian refugees	

In December 2016, staff from CDC completed an independent evaluation of the IOM program, with support from IOM and the Jordan and Lebanon NTPs. This paper will provide a descriptive analysis of the program to examine the program objectives of increasing TB case-finding (through increased screening and diagnosis, and conducting thorough contact investigations) and maximizing treatment success among Syrian refugees, examining 2013–2015 data in Jordan and 2015 data in Lebanon. It will also provide suggestions for program improvement.

## Methods

The evaluation consisted of a retrospective review of de-identified IOM and Jordan and Lebanon NTP line lists of TB cases and of individually investigated contacts (Lebanon only). Because this was an evaluation of a public health surveillance program with de-identified data and no perceived ethical risk to patients, no informed consent was obtained. This program review received non-research determination from CDC Center for Global Health, as well as written permission and participation of NTP leadership and staff in both countries.

TB notification rates for Jordan during 2013–2015 and for Lebanon in 2015 were calculated using UN Population Division midyear estimates for 2013–2015 as denominators [12]. Annual TB notification rates among Syrian refugees were calculated using midyear population estimates from UNHCR as denominators [13]. For 2015 data only, Syrian refugee TB cases were categorized by age group (aged < 5 years, 5–14 years, and ≥ 15 years), TB type (pulmonary versus extra-pulmonary), treatment outcome (cure or treatment completion versus other outcome) and in Jordan, by refugee camp status (residence in versus outside a refugee camp). In Lebanon, which has no official Syrian refugee camps, this distinction was not possible.

To evaluate where Syrian refugee TB cases were found, proportions of Syrian TB cases living in refugee camps were compared with proportions of the total Syrian population living in camps in Jordan in 2015 using chi-square analysis. Additionally, the TB notification rates among Syrian refugees in camps and outside of camps were compared, using midyear camp and non-camp UNHCR population estimates [13].

In Jordan during 2013–2015 and in Lebanon in 2015, annual numbers of Syrian refugees evaluated as household contacts of all TB cases, as well as numbers of incident Syrian TB cases found through these contact investigations, were used to calculate the number of evaluated Syrian refugee contacts needed to find one incident TB case.

To evaluate the completeness of contact investigations among Syrian refugees in Lebanon in 2015, the numbers of each Syrian index case’s total household contacts listed and investigated were compared. Bivariate Chi-square analysis of proportions, using covariates of TB type, sex, and age group, was done with binary outcome of the same or more contacts investigated than listed versus more listed than investigated. Such an analysis was not possible in Jordan, because although Jordan did document the total number of Syrian household contacts of index cases in 2015, IOM did not maintain a line list of contacts investigated for each index case.

To examine treatment outcomes among Syrian refugees facilitated by IOM engagement of community health workers, Chi-square analysis was done to compare proportions of treatment outcomes for 2013–2015 in Jordan and, in Lebanon, in 2014 (prior to full IOM support) and 2015. For 2015, bivariate analyses with covariates of sex and age group were conducted for both countries, and refugee camp status for Jordan, with treatment outcome as the dependent variable. For cell values of zero, Haldane correction was done to calculate odds ratios. Data analyses were performed in SAS Version 9.4 [14].

## Results

Syrians represented a substantial proportion of incident TB cases in both Jordan during 2013–2015 and Lebanon in 2015 (Table 2). In Jordan, the calculated TB notification rate for the entire population, including all foreign-born, increased from 4.5/100,000 population in 2013 to 5.5/100,000 population in 2015. The proportion of total notified TB cases among Syrian refugees decreased year-on-year, from 24.4% of cases in 2013, when Syrians refugees made up 6.8% of the country’s population, to 13.8% of cases in 2015, when Syrians made up 8.3% of the total population. Additionally, although the annual notification rate among Syrian refugees remained consistently higher than the overall notification rate, the Syrian TB notification rate declined in Jordan between 2013 and 2015 (Table 2).

**Table 2:** Notified incident TB cases and rates among general population and Syrian refugees, Jordan, 2013–2015, and Lebanon, 2015

**Jordan**			
Year	2013 n (%)	2014 n (%)	2015 n (%)
Notified incident TB cases (all forms)	324	379	421
Total population including foreign-born^a^	7,215,000	7,416,000	7,595,000
Calculated TB notification rate (notified cases/total population)	4.5/100,000	5.1/100,000	5.5/100,000
Notified incident TB cases among native Jordanians	179 (55.2)	216 (57.0)	196 (46.6)
Notified incident TB cases among foreign-born non-Syrians	66 (20.4)	91 (24.0)	167 (39.7)
Notified incident TB cases among Syrian refugees	79 (24.4)	72 (19.0)	58 (13.8)
Total Syrian refugee population^b^	491,365	604,868	629,128
Calculated TB notification rate among Syrian refugees (notified cases/total population) (95% CI)	16.1/100,000 (12.9–20.0)	11.9/100,000 (9.4–15.0)	9.2/100,000 (7.1–11.9)
**Lebanon**			
Year			2015 n (%)
Notified incident TB cases (all forms)			650
Total population including foreign-born^a^			5,851,000
Calculated TB notification rate (notified cases/total population)			11.1/100,000
Notified incident TB cases among native Lebanese			297 (45.7)
Notified incident TB cases among foreign-born non-Syrians			214 (32.9)
Notified incident TB cases among Syrian refugees			139 (21.4)
Total Syrian refugee population^b^			1,174,830
Calculated TB notification rate among Syrian refugees (notified cases/total population) (95% CI)			11.8/100,000 (10.0–14.0)

^a^Midyear population estimates 2013–2015 from UN Population Division

^b^Midyear population estimates from UNHCR data

In Lebanon, the TB notification rate for the entire population, including foreign-born, was 11.1/100,000 population in 2015. The proportion of total incident TB cases among Syrians was 21.4% of cases in 2015, when Syrians made up 20.1% of the total population, and the annual notification rate was not significantly higher than the overall national incidence rate.

Both Jordan and Lebanon had substantial proportions of total TB cases among non-Syrian foreign-born persons. These cases were primarily among migrant workers from high TB-burden countries.

In Jordan in 2015, the proportion of TB cases among Syrian refugee camp residents was significantly higher than the proportion of the total Syrian population residing in camps (Table 3). When expressed as a comparison of case notification rates, the rate of TB cases among Syrian refugee camp residents (15.8/100,000) was twice that of TB cases among Syrians not living in camps (7.9/100,000). An exploratory analysis of potential demographic differences between Syrian TB cases residing in and outside of camps showed no difference in sex or mean age in 2015.

**Table 3:** Proportions of TB cases found among Syrian refugees residing in and outside of refugee camps, Jordan, 2015

Year: 2015	Proportion residing in refugee camps in Jordan, n (%)	Proportion residing outside refugee camps in Jordan, n (%)	*P* value
Population in Jordan			
Refugee TB cases (*n* = 58)	17 (29.3)	41 (70.7)	**0.013** ^b^
Total refugee population (*n* = 629,128)^a^	107,517 (17.1)	521,611 (82.9)	
Calculated TB notification rate (notified cases/total population)	15.8/100,000	7.9/100,000	**0.015** ^b^
Refugee TB cases (*n* = 58) sex			
Male (*n* = 32)	7 (41.2)	25 (61.0)	0.17
Female (*n* = 26)	10 (58.8)	16 (39.0)	
Refugee TB cases (*n* = 58) mean age (95% CL)	35.5 (26.1–44.9)	35.5 (28.9–42.0)	1.00

^a^Midyear population estimates from UNHCR data

^b^Bolded text signifies *P* value less than 0.05

Between 2013 and 2015, the number of Syrian refugees annually evaluated for active TB as contacts of index TB cases in Jordan decreased from 220 evaluated in 2013 to 180 in 2015 (Table 4). In Lebanon in 2015, the number evaluated in 2015 was 450. In Jordan, the number needed to be evaluated among Syrian contacts to find one active TB case was 90 in 2015, and in Lebanon, it was 30.

**Table 4:** Numbers of Syrian refugees screened for TB as contacts, Jordan, 2013–2015, and Lebanon, 2015

**Jordan**			
Year	2013, n	2014, n	2015, n
Number of Syrian refugees screened for TB as contacts of index TB cases	220	120	180
Number of incident TB cases among Syrian refugees found through contact investigations	3	8	2
Number of Syrian refugee contacts screened to find one active TB case (Number of refugee contacts screened /number of incident cases found)	73.3	15.0	90.0
**Lebanon**			
Year			2015, n
Number of Syrian refugees screened for TB as contacts of index TB cases			450
Number of incident TB cases among Syrian refugees found through contact investigations			15
Number of Syrian refugee contacts screened to find one active TB case (Number of refugee contacts screened /number of incident cases found)			30.0

Examining linkage of Lebanon’s Syrian TB cases and investigated contacts showed that in 2015, among 126 Syrian index cases with household contacts listed, 98 (77.8%) had either agreement between the number of close contacts listed and the number of contacts investigated or had more contacts investigated than listed (Table 5). There was no significant difference between type of TB, sex, or age group in agreement between number of close contacts listed and the number of contacts investigated or more contacts investigated than listed. Of note, the 14 Syrian index cases age less than 15 (7 with age < 5 years, 7 with age 5–14 years) all had investigations for all contacts listed.

**Table 5:** TB contact investigations of Syrian index cases, Lebanon, 2015

Lebanon			
Year: 2015	Agreement between number of close contacts reported and number of contacts investigated or more contacts investigated than number reported, n (%)	Number of close contacts reported exceeded number of contacts investigated, n (%)	Odds ratio from bivariate analysis (95% CI)
Total Syrian index cases with household contacts listed (*n* = 126)	98 (77.8)	28 (22.2)	n/a
Type of TB			
Pulmonary (*n* = 94)	70 (74.5)	24 (25.5)	0.4 (0.1–1.3)
Extra-pulmonary (*n* = 32)	28 (87.5)	4 (12.5)	Ref
Sex			
Male (*n* = 74)	58 (78.4)	16 (21.6)	1.1 (0.5–2.5)
Female (*n* = 52)	40 (76.9)	12 (23.1)	Ref
Age group			
Less than 5 years (*n* = 7)	7 (100)	0 (0)	5.1 (0.3–91.4)
5–14 years (*n* = 7)	7 (100)	0 (0)	5.1 (0.3–91.4)
15 years or greater (*n* = 112)	84 (75.0)	28 (25.0)	Ref

**Fig. 1:**
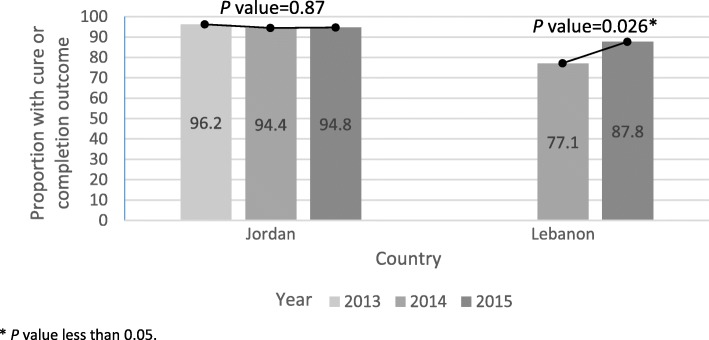
Proportions of Syrian TB cases with cure or completion treatment outcome by year, Jordan and Lebanon, 2013–2015

In Jordan, between 2013 and 2015, treatment cure or completion as outcome among Syrian refugees remained high, with all years above 94% cure or completion (Fig. 1). In contrast, in Lebanon between 2014 (prior to full IOM support) and 2015, the cure or completion rate rose from 77.1% in 2014 to 87.8% in 2015, a significant increase.

In Jordan, bivariate analysis showed no difference in the odds of having an outcome of cure or completion by sex, age group, or camp status in 2015, the only year with complete data (Table 6). In Lebanon in 2015, males had lower odds (Odds ratio: 0.3, 95% Confidence Interval (CI): 0.1–0.99) than females but age group had no difference in odds of an outcome of cure or completion.

**Table 6:** Numbers, proportions, and factors associated with TB treatment outcome of cure or completion among Syrian refugees, Jordan and Lebanon, 2015

**Jordan**			
**Year**	**Cure or completion outcome n (%)**	**Outcome other than cure or completion n (%)**	**Odds ratio from bivariate analysis (95% CI)**
2015	55 (94.8)	3 (5.2)	n/a
Sex			
Male	29 (90.6)	3 (9.4)	0.2 (0.01–3.2)
Female	26 (100)	0 (0)	Ref
Age group			
Less than 5 years	6 (100)	0 (0)	0.9 (0.04–19.9)
5–14 years	3 (100)	0 (0)	0.5 (0.02–12.4)
15 years or greater	46 (93.9)	3 (6.1)	Ref
Camp	16 (94.1)	1 (5.9)	0.8 (0.1–9.7)
Non-camp	39 (95.1)	2 (4.9)	Ref
Lebanon			
Year	Cure/Completion n (%)	Outcome other than cure or completion n (%)	Odds ratio from bivariate analysis (95% CI)
2015	122 (87.8)	17 (12.2)	n/a
Sex			
Male	68 (82.9)	14 (17.1)	**0.3 (0.1–0.99)** ^*^
Female	54 (94.7)	3 (5.3)	Ref
Age group			
Less than 5 years	8 (100)	0 (0)	2.6 (0.1–47.0)
5–14 years	8 (88.9)	1 (11.1)	0.9 (0.1–5.4)
15 years or greater	106 (86.9)	16 (13.1)	Ref

*Bolded text signifies *P* value less than 0.05

## Discussion

The arrival of Syrian refugees has had a substantial impact on Jordan and Lebanon NTPs. In 2013, TB cases among Syrian refugees made up 24.4% of all TB cases in Jordan, and in 2015, 13.8% of all TB cases in Jordan and 21.4% of all TB cases in Lebanon. The difference in proportion of TB cases among Syrian refugees between Jordan and Lebanon in 2015 may reflect unmeasured differences in demographics or TB rates of region of origin in Syria, among the Syrian refugees in each country. Syrian refugees in Jordan had a higher TB case notification rate than Jordan’s overall notification rate, assuming that UNHCR refugee population data capture the true population numbers, although it bears noting that the Syrian notification rate declined in Jordan between 2013 and 2015. This decline may in part be due to the possibility that Syrian TB cases found in 2013, soon after the IOM program of support began, reflect both prevalent as well as incident cases, whereas later years’ cases, after increased surveillance and case-finding, reflect only incident cases. This pattern aligns with changes through time in the Syrian refugee population in Jordan: during 2013, the population doubled, and thereafter stabilized [13]. It also aligns with changes through time in the proportion of the Syrian refugees living in camps: in midyear 2013, 34% of Syrian refugees in Jordan lived in camps, but this proportion declined to and stabilized at less than 20% thereafter [13]. Documentation of delay between onset of symptoms and diagnosis of TB may help elucidate whether notified cases are relatively old or new, but this case information is not collected by IOM or NTP.

A higher proportion of TB cases in both countries was among non-Syrian foreign-born persons coming from high-incidence countries than among Syrian refugees [15]. Thus, efforts to improve case-finding and treatment that focus only on Syrian refugees risk missing TB cases among other vulnerable populations, such as migrant workers. IOM now additionally supports both NTPs in case detection, treatment, and case referral among non-Syrian foreign-born persons (A. Galev, personal communication, 10 December 2017).

In 2013, when WHO estimated a TB incidence rate of 17 cases/100,000 population in Syria, Syrian refugee population data indicate an expected number of notified cases among Syrian refugees in Jordan of 84, close to the 79 cases notified that year [7]. In contrast, in 2015, when the WHO estimated incidence rate in Syria was 20 cases/100,000 population (95% CI 15–25 cases) and the Syrian refugee population was higher, the expected number of notified cases among Syrian refugees was 126 in Jordan (2.1 times the 58 notified cases) and 235 in Lebanon (1.7 times the 139 notified cases) [16]. In 2015, TB notification rates among Syrian refugees in both Jordan and Lebanon were less than the WHO-estimated incidence rate [16]. While this difference could reflect missing cases in Jordan and Lebanon in 2015, it may also be because TB incidence among Syrian refugees decreased through time, as fewer new arrivals came to host countries, and as the incidence among refugees with a more prolonged stay began to mirror host population incidence.

Syrian refugee TB cases in Jordan were disproportionately more likely to reside in camps than the general Syrian refugee population in 2015: 29% of Syrian TB cases lived in camps while only 17% of the Syrian population lived in camps, with a TB case notification rate among Syrians in camps twice that among Syrians outside of camps. This difference was not explained by consistent differences in sex and age between those residing in and outside of camps, and thus other factors may contribute to this difference. Crowded living conditions, as can occur in refugee camps, have been associated with TB transmission [17]. Alternatively, low socioeconomic status or malnutrition, which may be higher among refugees living in camps, are associated with TB prevalence or reactivation [18–21]. IOM screenings focus on pulmonary symptoms in camps, while community-based surveillance has historically been passive, with less developed active case-finding activities. This finding may also suggest that in Jordan in 2015, there were community TB cases missed, though the veracity of this finding is limited by the fact that other demographic data that may indicate systemic differences between camp-based and community-bases refugees were unavailable. Though Lebanon has no Syrian refugee camps, documentation of residence in informal settlements or collective shelters could help determine if a difference in case finding by residence also exists in Lebanon.

In reviewing the total numbers of Syrian refugees evaluated annually as contacts of TB cases in these two countries, there was no persistent trend in number evaluated to find an active TB case. The uptick in number evaluated as contacts in Jordan from 2014 to 2015 and the comparatively high number in Lebanon in 2015 corresponded in Jordan to the end of IOM’s conducting border screening for all new arrivals in 2014 and in Lebanon to the suspension of new refugee registration in 2015. Thus, the 2015 numbers perhaps reflect an IOM program shift from mass screenings of new arrivals to improving contact investigations among Syrians. Mass screening of TB symptoms among refugees remains an important strategy for active case finding, but in Jordan and Lebanon, where new arrivals have declined, thorough contact investigations may be more efficient in this population going forward.

In Lebanon, review of Syrian index cases with contact investigations in 2015 showed that just 78% of those cases with contacts listed had complete investigations, thus indicating room for improvement in contact investigations. Of note, all cases age less than 15 had complete investigations. A recent study in Jordan of contact investigations among Syrian refugee TB cases found a high proportion of contacts were investigated, but noted a need for increased attention to children under 5 [22]. In this evaluation, there were insufficient data on diagnostic tests or treatments provided to investigated contacts to evaluate the effectiveness of the investigations. An increased focus in Lebanon, and a maintained focus in Jordan, on management and documentation of contact investigations may help assess and improve the effectiveness of these investigations.

Treatment outcome of cure or completion among Syrian refugee TB cases was high in Jordan between 2013 and 2015, and it improved significantly in Lebanon from 2014 to 2015. In fact, in this population in 2015, Jordan exceeded and Lebanon approached the WHO End TB goal of 90% treatment success [23]. The improvement in Lebanon coincided with enhanced IOM support for staffing to provide patient follow-up. In Lebanon in 2015, males were less likely than females to attain an outcome of cure or completion. Similarly, in a case-control study among TB patients in Kenya, being male was independently associated with loss to follow-up [24]. Ensuring treatment success is essential to halt transmission of TB and to prevent the emergence of drug resistance, but this is especially difficult in transient populations such as refugees. In northeastern Kenya, camp-based refugees had 3.7 greater odds than host-community patients of having drug-resistant TB [25]. Although Jordan and Lebanon have seen very few multidrug-resistant TB (MDR-TB) cases among Syrian refugees during the examined time period, there is some concern that MDR-TB may be emerging in Syria, thus underscoring the need for continued assurance of treatment success [9].

This evaluation was subject to limitations. First, although we used the 2015 WHO estimated Syria incidence rate to calculate expected number of notified cases among Syrian refugees, the accuracy of that estimate may be affected by reporting limitations within the Syrian displacement crisis. Second, although the TB control strategy included the objective of increasing TB case-finding, this public health program evaluation, which used retrospective, surveillance data in the setting of an unstable population, could not address whether TB case-finding among Syrian refugees increased. Finally, because the evaluation lacked standardized baseline data on TB activities among Syrian refugees in the initial phases of the displacement crisis, and because it describes a relatively short timeframe, measurement of impact of the program on TB case-finding is difficult.

## Conclusions

The IOM program of support to Jordan and Lebanon NTPs for TB case-finding and treatment among Syrian refugees in Jordan and Lebanon provides critical services to this population. Recognizing that TB cases among non-Syrian foreign-born persons make up a substantial component of TB burden in these countries, the IOM program has expanded to provide services to this population. More community and health practitioner outreach for enhanced active case finding among community-based Syrian refugees in Jordan may improve TB case detection in populations outside of refugee camps. Thorough contact investigations need continued emphasis, including completely recording investigations, to find active TB cases and to advance the goal of providing TB preventive treatment for latent TB infection [26, 27].

Treatment success rates among Syrian refugees are high in Jordan and improved markedly in Lebanon, through IOM’s support to the NTP. Enhanced efforts to prevent loss to follow-up, including among males in Lebanon, are necessary. In addition, ongoing IOM and WHO regional initiatives in cross-border case surveillance and treatment programs for Syrian refugees fit with the current emphasis on transnational solutions for halting spread of TB [26, 28, 29]. Taken together, these efforts are necessary to reduce, and eventually eliminate, TB among this vulnerable population.

## Data Availability

The data collected belong to the respective countries’ NTPs and IOM, and thus are not publicly available. However, data are available from the authors upon reasonable request and with permission from the respective countries’ NTPs and IOM.

## Abbreviations

CDC: (United States) Centers for Disease Control and Prevention
IOM: International Organization for Migration
MDR-TB: Multidrug-resistant tuberculosis
NTP: National TB Program
UNHCR: United Nations High Commissioner for Refugees
WHO: World Health Organization
